# Characteristics, Management, and Outcomes of Acute Life-Threatening Asthma in Adult Intensive Care

**DOI:** 10.3390/clinpract14050149

**Published:** 2024-09-12

**Authors:** Adam J. R. Watson, Thomas Roe, Oliver Arscott, Charlotte Thomas, James Ward, Ryan Beecham, David Browning, Kordo Saeed, Ahilanandan Dushianthan

**Affiliations:** 1 General Intensive Care Unit, University Hospital Southampton NHS Foundation Trust, Southampton SO16 6YD, UK; 2 Clinical & Experimental Sciences, Faculty of Medicine, University of Southampton, Southampton SO16 6YD, UK; 3 Department of Infection, University Hospital Southampton NHS Foundation Trust, Southampton SO16 6YD, UK; 4 Perioperative & Critical Care Theme, NIHR Southampton Biomedical Research Centre, Southampton SO16 6YD, UK

**Keywords:** asthma, intensive care unit, life-threatening, near fatal, ventilation

## Abstract

Background: There is limited evidence regarding the management of acute life-threatening asthma in intensive care units (ICUs), and few guidelines have details on this. We aimed to describe the characteristics, management, and outcomes of adults with life-threatening asthma requiring ICU admission. Methods: In this single-centre retrospective observational study, we included consecutive adults with acute asthma requiring ICU admission between 1 January 2016 and 31 December 2023. Our primary outcome was requirement for invasive mechanical ventilation (IMV). Results: We included 100 patients (median age 42.5 years, 67% female). The median pH, PaCO_2_, and white cell count (WCC) on ICU admission were 7.37, 39 mmHg, and 13.6 × 109/L. There were 30 patients (30%) who required IMV, and the best predictors of IMV requirement were pH (AUC 0.772) and PaCO_2_ (AUC 0.809). In univariate analysis, IMV requirement was associated with both increasing WCC (OR 1.14) and proven bacterial infection (OR 8.50). A variety of respiratory support strategies were utilised, with 38 patients (38%) receiving only non-invasive respiratory support. Conclusions: Our data highlight key characteristics which may be risk factors for acute asthma requiring ICU admission and suggest that pH, PaCO_2_, and WCC are prognostic markers for disease severity. Our overall outcomes were good, with an IMV requirement of 30% and a 28-day mortality of 1%.

## 1. Introduction

Asthma is a chronic respiratory disease characterised by inflammation and reversible airflow obstruction [1]. The healthcare burden from asthma is significant, with nearly half a million global asthma-related deaths in 2019 [2]. In the United Kingdom (UK), over 20,000 adults require hospital admission for acute exacerbations of asthma per annum, with a median hospital stay of two days [3]. Furthermore, in the most severe life-threatening exacerbations, representing approximately 1–10% of hospitalised patients [4,5], admission to an intensive care unit (ICU) is required. In most healthcare systems, an ICU admission facilitates the use of additional advanced pharmacological therapies and respiratory support modalities [6]. The prevalence of acute asthma in the ICU case mix is 1–2% according to historic data, of whom up to half might require invasive mechanical ventilation (IMV) [5,7,8].

However, there is little evidence about the management of asthma in an ICU setting. The majority of studies describing the characteristics and outcomes of adults with acute life-threatening asthma are historic [5,6,7,8,9] but generally report low mortality rates and relatively short ICU admissions. More recent data hint at an increasing use of non-invasive ventilation, which continues to be a controversial but potentially beneficial treatment [10,11,12]. Overall, high-quality evidence to guide treatment decisions is lacking [6], and as such most guidelines have few details on the management of acute life-threatening asthma in the ICU [13,14,15].

We therefore aimed to describe a cohort of adults admitted to our ICU with acute life-threatening asthma. Our primary objective was to report the characteristics, treatment, and outcomes of this patient group. Our secondary objective was to identify any risk factors for adverse outcomes in the ICU.

## 2. Methods

### 2.1. Study Design and Setting

In this single-centre retrospective observational study, we included consecutive adults with acute asthma requiring ICU admission. The study data were collected for the period between 1 January 2016 and 31 December 2023. This is part of a large cohort study (CRIT-CO) investigating outcomes for critically ill patients in ICUs. The study was sponsored by the University Hospital Southampton NHS Foundation Trust (RHM CRI 0370) and approved by the Health Research Authority and Health and Care Research Wales (HCRW) (IRAS 232922, approval date: 26 November 2018). The manuscript complies with STROBE guidelines [16], and no identifiable patient data are presented here.

### 2.2. Inclusion and Exclusion Criteria

In our ICU, patients’ diagnoses are contemporaneously recorded during ICU admission. We searched these and included adult patients with a clinical diagnosis of acute severe or life-threatening exacerbation of asthma. We excluded patients with irretrievable patient records or other acute respiratory pathology (e.g., pneumonia) as an admission diagnosis. For example, a patient with a “severe infective exercitation of asthma” would have been included, whereas a patient with “pneumonia and asthma exacerbation” would have been excluded. Of note, in our hospital, most patients with exacerbations of COPD are managed on a dedicated respiratory high-dependency unit (HDU), so were not eligible as they were not admitted to our ICU.

### 2.3. Data Collection

Anonymised patient data were retrieved from our electronic patient records (MetaVision, iMDsoft, Tel Aviv, Israel). The data collected included patient demographic information (age, sex, ethnicity, Body Mass Index, and smoking status), co-morbidities and frailty (described using Charlson’s Comorbidity Index, CCI [17], and Clinical Frailty Score, CFS [18]), asthma characteristics (previous admissions to ICU and regular medications), and clinical presentation (laboratory values, physiological observation, and microbiology data). Patients were categorised as exhausted if their medical notes recorded this, whilst altered mental status was defined as a Glasgow Coma Score < 15. We also recorded pharmacological treatment and respiratory support received. The primary outcome reported is requirement for invasive mechanical ventilation (IMV). The secondary outcomes are requirement for non-invasive respiratory support (NIRS; defined as non-invasive ventilation, continuous positive airway pressure, or high-flow nasal oxygen), requirement for extra-corporal membrane oxygenation or carbon dioxide removal (ECMO or ECCO_2_R), ICU length of stay, in-ICU mortality, and 28-day mortality.

### 2.4. Statistical Analysis

Our data are reported using conventional descriptive statistics, with categorical data presented as numbers (percentage of whole cohort). We used the Kolmogorov–Smirnov test to assess continuous data for normality, and as our dataset was generally non-normally distributed, we present continuous variables as median (inter-quartile range; IQR). We classify patients by maximum level of respiratory support received (IMV vs. NIRS vs. no support) and compare between these groups using the Chi-squared test for categorical and Kruskal–Wallis one-way ANOVA for continuous data. Patients with partially incomplete data (e.g., admission blood gas results) were excluded from specific analyses as needed. The ability of variables to predict outcomes was investigated using univariate logistic regression and linear regression, as well as receiver operating characteristic (ROC) curve analysis. We report the associated Odds Ratios (ORs) and area under the curve (AUC) for these variables. A modified survival curve was used to display the proportion of patients that remained admitted to the ICU over time. We used SPSS v28 (IBM Corp., Armonk, NY, USA) for our analysis, with a *p*-value of <0.05 taken to be significant.

## 3. Results

**Figure 1:**
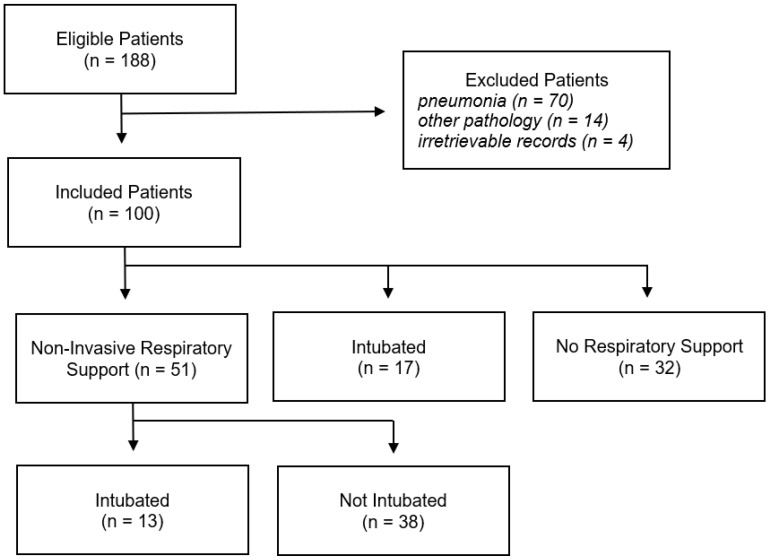
Flow diagram of eligible, included, and excluded patients.

There were 188 eligible patients admitted to our ICU (Figure 1). Of these, 84 had another acute respiratory pathology as their primary diagnosis, so were excluded. A further 4 patients with irretrievable patient records were also excluded.

### 3.1. Patient Characteristics

We included 100 patients with a median age of 42.5 yeas (IQR 32.0–53.3, 67% female, 88% white ethnicity). The median Charlson Comorbidity Index and Clinical Frailty Scale scores were 0 (IQR 0–2) and 2 (IQR 1–3), respectively. Whilst 48 patients (48%) were current or former smokers, only 9 patients (9%) reported COPD as an additional co-morbidity. Of note, 32 patients (32%) had previously required ICU admission for acute severe asthma, whilst 15 (15%) and 13 (13%) patients, respectively, were prescribed long-term oral steroids or biologic therapies for asthma control. There were some differences in patient characteristics according to the maximum level respiratory support required, particularly between patients who received only NIRS compared to those who either required IMV or no respiratory support (Table 1). Specifically, patients who received only NIRS reported the highest prevalence of COPD (21%, *p* < 0.001), fewer previous ICU admissions related to asthma (13%. *p* = 0.007), and were less likely to be prescribed biologic therapy (5%, *p* = 0.030).

### 3.2. Clinical Presentation

On admission to the ICU, there were 70 patients (70%) felt to be exhausted, whilst 29 (29%) had altered mental status. The median pH, PaCO_2_, and PaO_2_/FiO_2_ ratio were 7.37 (IQR 7.30–7.41), 39 mmHg (IQR 33–47), and 236 mmHg (IQR 155–313), respectively. In addition, median respiratory and heart rates were 28 (IQR 20–34) and 112 (IQR 100–126), whilst the overall median white cell count (WCC) and C-reactive protein (CRP) were 13.6 × 10^9^/L (IQR 10.4–18.6) and 22 mg/L (IQR 8–77), respectively. Furthermore, 26 patients (26%) subsequently had a proven viral or bacterial respiratory tract infection during their ICU admission (Table 1). These patients had a higher median admission WCC (20.3 vs. 13.3, *p* = 0.047); however, there was no difference in admission CRP between patients who had a proven infection and those who did not (*p* = 0.516). The identified respiratory tract pathogens were rhinovirus (*n* = 8, 8%), *Staphylococcus aureus* (*n* = 5, 5%), metapneumovirus (*n* = 4, 4%), parainfluenza virus type 3 (*n* = 3, 3%), *Pseudomonas aeruginosa* (*n* = 2, 2%), *Hemophilus influenzae* (*n* = 2, 2%), Influenza A (*n* = 1, 1%), respiratory syncytial virus (*n* = 1, 1%), adenovirus (*n* = 1, 1%), *Moraxella catarrhalis* (*n* = 1, 1%), and *Escherichia coli* (*n* = 1, 1%).

There were notable differences in presentation according to maximum level of respiratory support required (Table 1). Firstly, patients requiring IMV had the highest prevalence of exhaustion (87%, *p* = 0.001) and altered mental status (67%, *p* < 0.001) on ICU admission. Secondly, these patients had the lowest pH (7.28, *p* < 0.001) and highest PaCO_2_ (53 mmHg, *p* < 0.001), although there was no difference in the PaO_2_/FiO_2_ ratio between groups (*p* = 0.823). Furthermore, patients requiring IMV had the highest initial WCC (17.3 × 10^9^/L, *p* = 0.003), and a greater proportion subsequently had a proven bacterial respiratory tract infection (20%, *p* = 0.003).

### 3.3. Patient Outcomes

There were 30 patients (30%) who required IMV. On ICU admission, we initially managed 51 patients (51%) with NIRS, and of these, 13 subsequently required IMV (13%, ‘failed NIRS’), whilst the other 38 did not (38%, ‘succeeded NIRS’). There were 17 patients (17%) who received immediate IMV on ICU admission. If patients who ‘succeeded’ or ‘failed’ NIRS were compared, there was no differences in median pH (*p* = 0.130), PaCO_2_ (*p* = 0.138), PaO_2_/FiO_2_ ratio (*p* = 0.218), or WCC (*p* = 0.486) on ICU admission.

**Figure 2:**
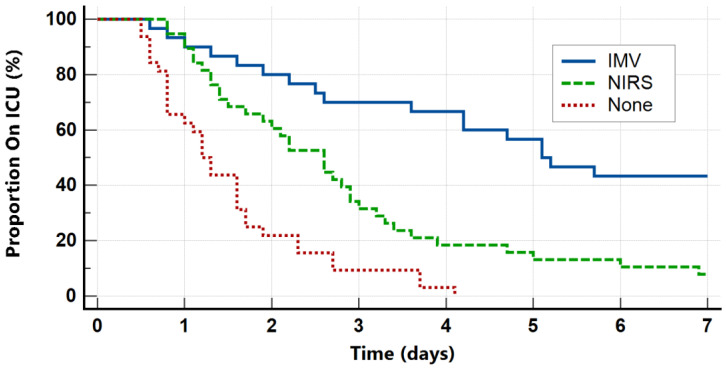
Proportion of patients admitted to ICU over time according to maximum level of respiratory support.

The overall median ICU length of stay was 2.3 days (IQR 1.3–4.4). Furthermore, the length of stay was strongly associated with the maximum level of respiratory support (Figure 2), with patients requiring IMV having the longest median ICU stays (5.1 days, *p* < 0.001). The overall prevalence of ECMO or ECCO2R use was 2%, whilst in-ICU and 28-day mortality were both 1%.

### 3.4. Outcome Predictors

We conducted univariate logistic and linear regression analyses to investigate variables associated with the requirement for IMV or ICU length of stay. The presence of altered mental status or bacterial infection was strongly associated with requiring IMV, with univariate ORs of 13.60 (95% CI 4.83–38.06, χ^2^ = 28.53, df = 1, *p* < 0.001) and 8.50 (95% CI 1.61–45.00, χ^2^ = 7.57, df = 1, *p* = 0.012), respectively. In addition, increasing PaCO_2_ and WCC, as well as decreasing pH, were also associated with IMV requirement; however, respiratory rate, heart rate, and PaO_2_/FiO_2_ ratio were not (Table 2). Furthermore, ICU length of stay had univariate linear relationships with pH (β = −30, R^2^ = 0.19, F = 21.86, *p* < 0.001), PaCO_2_ (β = 0.18, R^2^ = 0.19, F = 22.5, *p* < 0.001), and WCC (β = 0.3, R^2^ = 0.076, F = 7.61, *p* = 0.007).

**Figure 3:**
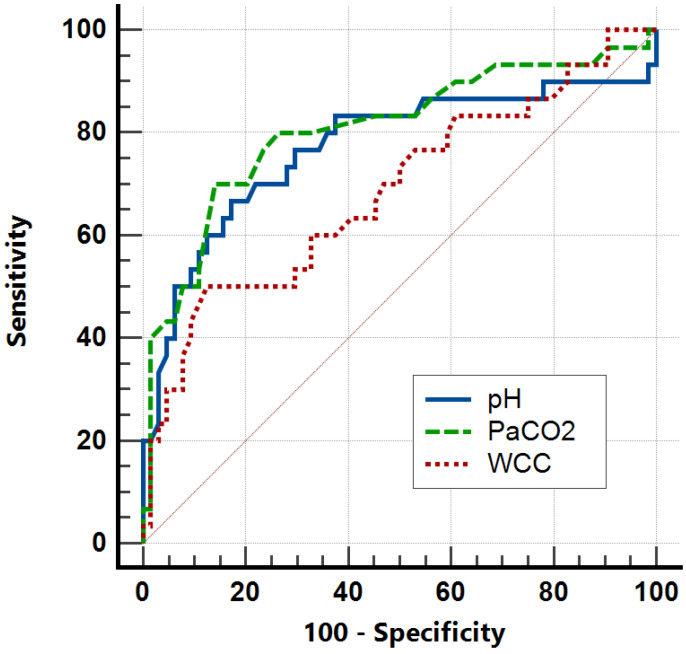
AUROC analysis for pH, PaCO_2_, and WCC on ICU admission as predictors of IMV requirement. Red solid line represents random predictor.

The best biochemical predictors of requiring IMV were decreased pH and increased PaCO_2_ (Figure 3), with AUCs of 0.772 (95% CI 0.109–0.347, *p* < 0.001) and 0.809 (95% CI 0.705–0.914, *p* < 0.001), respectively. Furthermore, increased WCC also moderately predicted IMV requirement, with an AUC of 0.689 (95% CI 0.569–0.810, *p* = 0.002). A cutoff of PaCO_2_ > 45 mmHg maximised Youden’s Index to 0.566, with an associated sensitivity of 70.0 (95% CI 50.6–85.3) and specificity of 86.6 (95% CI 76.3–96.7). Furthermore, a cutoff of pH < 7.32 maximised Youden’s Index to 0.503, with an associated sensitivity of 63.3 (95% CI 43.9–80.1) and specificity of 83.6 (95% CI 72.5–91.5). In contrast, a PaCO_2_ of < 35 mmHg had a sensitivity of 58.6 (95% CI 38.9–76.5) and a specificity of 80.9 (95% CI 69.5–89.4) for predicting no requirement for any respiratory support, whilst a WCC < 11 × 109/L had a sensitivity of 37.5 (95% CI 21.1–56.3) and a specificity of 76.1 (95% CI 64.1–85.7) for the same outcome.

### 3.5. Management

We report widespread use of nebulised bronchodilators, corticosteroids, and IV magnesium (Table 3). In addition, 62 patients (62%) received IV aminophylline, 28 (28%) received IV salbutamol, 21 (21%) received IV adrenaline, and 20 (20%) received IV ketamine. The use of these advanced bronchodilator pharmacological therapies was most prevalent amongst patients receiving IMV (Table 3). Of note, 64 patients (64%) received antibiotics, with no difference in admission WCC (*p* = 0.999) or CRP (*p* = 0.388) between those who did and did not receive antibiotics. We also managed 27 patients (27%) with mucolytics, including n-acetylcysteine (*n* = 9, 9%), carbocisteine (*n* = 18, 18%), and hypertonic saline (*n* = 10, 10%).

There were 24 patients (24%) who received non-invasive ventilation (NIV) as their first mode of respiratory support, with a median NIV duration of 0.7 days (IQR 0.1–1.7). The median NIV start settings were IPAP 14 cmH_2_O (IQR 13–18) and EPAP 6 cmH_2_O (IQR 5–8), generating a median tidal volume of 6.7 mL/kg (IQR 4.6–8.3). In addition, 23 patients (23%) received high-flow nasal oxygen (HFNO) as their first respiratory support, with a median start FiO_2_ of 0.60 (IQR 0.48–0.70) and an airflow of 45 L/min (IQR 39–53). A further 4 patients (4%) initially received continuous positive airway pressure (CPAP) with a median pressure of 7 cmH_2_O (IQR 5–9).

There were 30 patients (30%) who received invasive mechanical ventilation (IMV) at any point, with a median IMV duration of 5.0 days (IQR 1.4–8.3). The most common starting mode was volume-targeted adoptive pressure ventilation (*n* = 18, 60%), whilst the median start PEEP was 5 cmH_2_O (IQR 5–9), generating a median tidal volume of 5.8 mL/kg (IQR 4.5–7.8) with a I:E ratio of 1:2.6 (IQR 1:2.1–1:3.0).

## 4. Discussion

We report here the characteristics and outcomes of adults with acute life-threatening asthma requiring ICU admission. This study highlights specific risk factors for life-threatening asthma, including smoking history and previous ICU admission (Table 1), as well as confirming the significance of admission pH and PaCO_2_ in this patient cohort (Table 2). Furthermore, our data also suggest that WCC and the development of respiratory tract infection are important factors in predicting mechanical ventilation. We are also amongst the first to describe in detail the management of asthmatic ICU patients (Table 3), with frequent use of NIRS alongside advanced pharmacological therapies, ultimately leading to good ICU outcomes.

Asthmatic adults on ICUs are an understudied patient group, with limited evidence leading to contrasting guidance [19], and our data provide an update to historic studies of a similar nature [11,20]. However, there is significant heterogeneity in ICU practice globally, and lower ICU bed available in the United Kingdom is associated with a higher severity of illness on ICU admission [21]. Furthermore, we did not include patients managed on our hospital’s Respiratory High Care Unit, which principally manages COPD exacerbations using NIRS. As a result, our findings likely reflect acute asthma at the uppermost end of severity, including fewer patients with COPD/asthma overlap, and may therefore be most relevant to ICU clinicians from the United Kingdom or similar healthcare systems. We use the maximum level of respiratory support as a proxy for disease severity, as requirement for IMV is typically felt to reflect patients with ‘near fatal’ asthma exacerbations [22,23].

Our data highlight various characteristics which are common amongst patients requiring ICU admission (Table 1). The high prevalence of females and smokers in our cohort are well recognised risk factors for severe asthma [24], although otherwise our cohort is relatively young and healthy. However, nearly one-third of patients reported a previous asthma-related ICU admission, whilst 13 and 15% of patients were prescribed long-term oral steroids or biologic therapy. These findings suggest that ‘difficult to control’ asthma, as reflected by previous life-threatening exacerbations or the use of drugs of last resort, is likely to be a significant risk factor for further ICU admissions. We also demonstrate some differences in patient characteristics according to disease severity, which hints to the mindset of ICU clinicians (Table 1). For example, patients who ultimately did not require any respiratory support had better biochemical and clinical markers of disease severity on ICU admission but had the highest prevalence of previous ‘asthma’ ICU admissions, oral steroids, and biologic therapies. This suggests that these patients were possibly perceived to be so ‘high risk’ that ICU admission was warranted, even if just for monitoring. In contrast, patients who received NIRS, but did not progress to requiring IMV, had the lowest prevalence of previous ‘asthma’ ICU admissions, oral steroids, and biologic therapies, which may suggest the absence of these ‘risk factors’ predicts a positive response to NIRS.

The outcomes of asthmatic patients admitted to our ICU are good (Table 1). Overall, 30% of patients required IMV, which is comparable to previous studies [24], and we report a 28-day mortality of only 1%. Furthermore, the median ICU length of stay was 2.3 days, which is again consistent with historic data [7,20], and as expected, the length of stay was significantly longer in patients who required IMV (Figure 2). Our data also highlight various predictors of disease severity and poor outcomes. Both higher PaCO_2_ and lower pH are associated with requirement for IMV and ICU length of stay, whilst exhaustion or altered mental status also substantially increased the odds of requiring IMV (Table 2). These markers of disease severity have been previously reported and are reflected in some guidelines [13]. Of note, we also found that a high WCC on ICU admission both increased the odds of requiring IMV and was associated with ICU length of stay. Although WCC is not commonly used as a prognostic marker in the context of acute asthma, previous studies have found higher WCCs are associated with longer ICU admissions as well as the requirement for IMV or ECMO [25,26]. In this study, a normal WCC on ICU admission is moderately specific but not sensitive for not requiring any respiratory support, which, if combined with other clinical features and prognostic markers, could support decisions regarding ICU admission. Future work may consider investigating the prognostic importance of eosinophil count or IgE levels.

Our management of acute life-threatening asthma includes both advanced pharmacological therapies and different respiratory support modalities (Table 3). The initial widespread use of bronchodilators, steroids, and intravenous magnesium in our ICU is consistent with guidelines [13]. However, in contrast to previous studies [9], it appears that we use advanced intravenous pharmacological therapies (for example, IV salbutamol, IV adrenaline, or IV ketamine) more frequently. Although the available high-quality evidence to support these interventions is limited [27,28], few other options are available to ICU clinicians for the most severe exacerbations [6]. We managed 51% of patients with NIRS on ICU admission, whilst 24% specifically received NIV initially. The use of NIV for acute asthma remains controversial, although some recent evidence suggests an association with improved outcomes [11]. In our study, 25% of patients managed with NIRS on ICU admission subsequently required IMV, and there was no difference in initial biochemical markers of disease severity between patients who ‘succeeded’ or ‘failed’ on NIRS. The difficulties of ventilating patients with asthma are well described and beyond the scope of this study, although our findings further highlight the challenges of selecting the most appropriate respiratory support modality.

### Strengths and Limitations

Our study has several limitations. We have already discussed the heterogeneity in ICU practice globally and how this may affect the interpretation of our findings. As a single-centre retrospective observational study, our sample size was limited, and this, combined with a large number of confounding variables, meant we chose not to attempt multivariate analysis. Although we excluded patients with other acute respiratory pathology, some heterogeneity in our cohort may persist. Furthermore, retrospective data collection meant that accurately recording subjective markers of disease severity (e.g., exhaustion, and wheeze) was difficult, and thus we relied on more objective measures such as respiratory support received or biochemical variables. Moreover, we did not collect the prehospital use and their dosage of beta-2 agonists, corticosteroids, if the patient had any anti-IgE therapies, or details of ventilator strategy (e.g., pressure settings). Finally, we have been unable to describe complications of acute asthma or its management (for example, pneumothoraxes or arrhythmias). Nevertheless, we believe our study usefully describes the cohort of adults with acute life-threatening asthma. These data begin to help fill the research gap regarding the ICU management of asthma and highlight a number of areas for future research.

## 5. Conclusions

We report here the characteristics, management, and outcomes of adults with acute life-threatening asthma requiring ICU admission. Our data highlight key characteristics which may be risk factors for ICU admission and suggest that low pH (AUC 0.772), high PaCO_2_ (AUC 0.809), and increased WCC (AUC 0.689) are key markers for disease severity in the ICU. These findings may support clinicians with decisions about place of care and treatment strategies, as some patients with favourable biochemistry may be unlikely to require IMV. The outcomes of patients are generally good, with a median ICU length of stay of 2.3 days and 1% mortality at 28 days. Future research should clarify the importance of WCC as a prognostic marker or investigate whether specific treatments such as NIV are beneficial in this cohort.

## Figures and Tables

**Table 1:** Patient characteristics, presentation, and outcomes according to maximum respiratory support.

	All Patients (*n* = 100)	Invasive Mechanical Ventilation (*n* = 30)	Non-Invasive Respiratory Support (*n* = 38)	No Respiratory Support (*n* = 32)	*p*
**Patient Characteristics**					
Age (years)	42.5 (32.0–53.3)	43.0 (32.0–50.0)	46.0 (32.5–55.8)	41.0 (30.3–50.0)	0.573
Female sex	67 (67)	20 (70)	26 (68)	20 (63)	0.869
White ethnicity	88 (88)	27 (90)	34 (89)	27 (84)	0.745
Body Mass Index (kg/m^2^)	27.7 (24.0–34.6)	27.6 (23.9–37.1)	29.1 (24.1–34.6)	27.2 (23.7–33.3)	0.755
Charlson CI	0 (0–2)	0 (0–3)	2 (0–3)	0 (0–1)	0.064
Clinical Frailty Scale	2 (1–3)	2 (1–4)	2 (1–4)	1 (1–3)	0.199
Current smoker	22 (22)	7 (23)	9 (24)	6 (19)	0.865
Former smoker	26 (26)	8 (27)	9 (24)	8 (25)	0.961
COPD	9 (9)	0 (0)	8 (21)	1 (3)	<0.001
**Asthma Characteristics**					
Previous ICU admission	32 (32)	13 (43)	5 (13)	14 (44)	0.007
Long-term oral steroids	15 (15)	5 (17)	4 (11)	6 (19)	0.314
Biologic therapy	13 (13)	3 (10)	2 (5)	7 (22)	0.030
**Respiratory Data**					
Respiratory rate (per min)	28 (20–34)	29 (17–37)	30 (25–35)	24 (19–32)	0.099
Heart rate (per min)	112 (100–126)	117 (102–130)	114 (106–126)	108 (94–123)	0.274
Clinically exhausted	70 (70)	26 (87)	28 (74)	16 (50)	0.001
Altered mental status	29 (29)	20 (67)	8 (21)	1 (3)	<0.001
pH	7.37 (7.30–7.41)	7.28 (7.21–7.36)	7.38 (3.35–7.42)	7.39 (7.36–7.43)	<0.001
PaO_2_/FiO_2_ ratio (mmHg)	236 (155–313)	209 (129–419)	241 (158–314)	245 (208–274)	0.823
PaCO_2_ (mmHg)	39 (33–47)	53 (43–69)	39 (35–45)	33 (30–38)	<0.001
Bicarbonate (mmol/L)	22.9 (19.9–25.9)	24.7 (22.7–27.2)	23.0 (19.9–26.6)	20.5 (18.1–22.9)	<0.001
Base excess (mmol/L)	−2.7 (−4.7–−0.1)	−3.2 (−4.5–−0.6)	−1.8 (−4.9–0.3)	−3.5 (−5.1–−1.9)	0.284
Lactate	2.2 (0.9–4.4)	1.2 (0.7–2.3)	1.9 (0.9–3.8)	4.2 (2.2–5.5)	0.001
**Microbiology Data**					
White cell count (×10^9^/L)	13.6 (10.4–18.6)	17.3 (12.6–22.8)	14.5 (10.9–17.0)	11.5 (10.1–13.8)	0.003
Neutrophil count (×10^9^/L)	11.7 (8.8–15.7)	14.4 (10.8–19.0)	12.0 (8.8–14.9)	10.0 (8.7–12.1)	0.022
Lymphocyte count (×10^9^/L)	0.8 (0.6–1.3)	1.0 (0.6–1.4)	0.8 (0.6–1.1)	0.7 (0.6–1.2)	0.636
NLR	14.0 (7.8–23.4)	13.8 (7.3–31.6)	14.8 (9.0–23.5)	12.9 (7.3–17.9)	0.593
CRP (mg/L)	22 (8–77)	25 (11–82)	41 (9–96)	11 (5–27)	0.028
Proven viral infection	18 (18)	6 (20)	9 (24)	3 (9)	0.112
Proven bacterial infection	8 (8)	6 (20)	2 (5)	0 (0)	0.003
**Patient Outcomes**					
ICU length of stay (days)	2.3 (1.3–4.4)	5.1 (2.5–9.4)	2.6 (1.4–3.4)	1.3 (0.8–1.8)	<0.001
ECMO/ECCO_2_R	2 (2)	2 (6)	0 (0)	0 (0)	0.029
In-ICU mortality	1 (1)	1 (3)	0 (0)	0 (0)	0.125
Twenty-eight-day mortality	1 (1)	1 (3)	0 (0)	0 (0)	0.125

**Abbreviations**: Charlson CI, Charlson’s Comorbidity Index; COPD, chronic obstructive pulmonary disease; ICU, intensive care unit; NLR, neutrophil/lymphocyte ratio; CRP, C-reactive protein; ECMO, extracorporeal membrane oxygenation; ECCO_2_R, extra-corporeal carbon dioxide removal. **Footnotes**: Continuous data are presented as median (inter-quartile range) and categorical data as number (percentage). We compared the three sub-groups using Chi-squared and Kruskal–Wallis one-way ANOVA tests, with *p* values representing results of this comparison.

**Table 2:** Univariate ORs and AUCs for variables associated with requirement for IMV.

Predictor Variable	Univariate OR (95% CI, *p*)	AUC (95% CI, *p*)
Respiratory rate	1.01 (0.97–1.06, 0.637)	0.513 (0.370–0.657, 0.855)
Heart rate	1.01 (0.99–1.03, 0.595)	0.539 (0.406–0.67, 0.569)
Exhaustion	3.84 (1.21–12.24, 0.023)	0.619 (0.505–0.733, 0.041)
Altered mental status	13.56 (4.83–38.06, <0.001)	0.769 (0.658–0.880, <0.001)
pH (per 0.1)	0.33 (0.19–0.56, <0.001)	0.772 (0.676–0.851, <0.001)
PaCO_2_ (mmHg)	1.08 (1.04–1.13, <0.001)	0.809 (0.705–0.914, <0.001)
PaO_2_/FiO_2_ ratio	1.00 (0.99–1.00, 0.186)	0.460 (0.316–0.604, 0.584)
Lactate (mmol/L)	0.71 (0.55–0.92, 0.011)	0.676 (0.574–0.768, 0.002)
WCC (×10^9^/L)	1.14 (1.05–1.23, 0.001)	0.689 (0.569–0.810, 0.002)
CRP (mg/L)	1.00 (0.99–1.01, 0.254)	0.558 (0.430–0.686, 0.375)
Viral infection	1.21 (0.41–3.59, 0.734)	0.514 (0.389–0.639, 0.823)
Bacterial infection	8.50 (1.61–45.00, 0.012)	0.586 (0.457–0.714, 0.192)

**Abbreviations:** WCC, white cell count; CRP, C-reactive protein.

**Table 3:** Treatment received according to maximum level of respiratory support required.

	All Patients (*n* = 100)	Invasive Mechanical Ventilation (*n* = 30)	Non-Invasive Respiratory Support (*n* = 38)	No Respiratory Support (*n* = 32)	*p*
**Pharmacological Treatment**					
Nebulised Bronchodilators	100 (100)	100 (100)	100 (100)	100 (100)	1.000
Corticosteroids	95 (95)	29 (97)	36 (95)	30 (94)	0.593
Antibiotics	64 (64)	23 (77)	26 (68)	14 (44)	0.005
Mucolytics	27 (27)	16 (53)	8 (21)	3 (9)	<0.001
IV Magnesium	96 (96)	30 (100)	35 (92)	30 (94)	0.125
IV Aminophylline	62 (62)	24 (80)	23 (61)	15 (49)	0.007
IV Salbutamol	28 (28)	13 (43)	6 (16)	9 (28)	0.012
IV Adrenaline	21 (21)	17 (57)	2 (5)	2 (6)	<0.001
IV Ketamine	20 (20)	17 (57)	1 (3)	2 (6)	<0.001
Inhaled Isoflurane	4 (4)	4 (13)	0 (0)	0 (0)	0.002
**First Respiratory Support**					
HFNO	23 (23)	6 (20)	17 (45)	0 (0)	<0.001
CPAP	4 (4)	1 (3)	3 (8)	0 (0)	0.090
NIV	24 (24)	6 (20)	18 (47)	0 (0)	<0.001
IMV	17 (17)	17 (57)	0 (0)	0 (0)	<0.001

**Abbreviations**: HFNO, high-flow nasal oxygen; CPAP, continuous positive airway pressure; NIV, non-invasive ventilation; IMV, invasive mechanical ventilation. **Footnotes**: Categorical data are presented as number (percentage). We compared between groups using the Chi-squared test.

## Data Availability

The data presented in this study are available on request from the corresponding author.
